# Protein Translation Enzyme lysyl-tRNA Synthetase Presents a New Target for Drug Development against Causative Agents of Loiasis and Schistosomiasis

**DOI:** 10.1371/journal.pntd.0005084

**Published:** 2016-11-02

**Authors:** Arvind Sharma, Manmohan Sharma, Manickam Yogavel, Amit Sharma

**Affiliations:** Molecular Medicine Group, International Centre for Genetic Engineering and Biotechnology (ICGEB), New Delhi, India; Academy of Sciences of the Czech Republic, CZECH REPUBLIC

## Abstract

Helminth parasites are an assemblage of two major phyla of nematodes (also known as roundworms) and platyhelminths (also called flatworms). These parasites are a major human health burden, and infections caused by helminths are considered under neglected tropical diseases (NTDs). These infections are typified by limited clinical treatment options and threat of drug resistance. Aminoacyl-tRNA synthetases (aaRSs) are vital enzymes that decode genetic information and enable protein translation. The specific inhibition of pathogen aaRSs bores well for development of next generation anti-parasitics. Here, we have identified and annotated aaRSs and accessory proteins from *Loa loa* (nematode) and *Schistosoma mansoni* (flatworm) to provide a glimpse of these protein translation enzymes within these parasites. Using purified parasitic lysyl-tRNA synthetases (KRSs), we developed series of assays that address KRS enzymatic activity, oligomeric states, crystal structure and inhibition profiles. We show that *L*. *loa* and *S*. *mansoni* KRSs are potently inhibited by the fungal metabolite cladosporin. Our co-crystal structure of *Loa loa* KRS-cladosporin complex reveals key interacting residues and provides a platform for structure-based drug development. This work hence provides a new direction for both novel target discovery and inhibitor development against eukaryotic pathogens that include *L*. *loa* and *S*. *mansoni*.

## Author Summary

The fungal metabolite cladosporin is a potent and selective inhibitor of the malaria parasite protein translation machinery enzyme lysyl-tRNA synthetase (KRS). Our computational annotations of parasitic aaRSs from *Loa loa* and *Schistosoma mansoni* provide catalogs of these enzymes that drive parasitic protein translation. We have studied the drug inhibition of KRSs from two neglected tropical worm parasites *L*. *loa* and *S*. *mansoni*. Our results show that these single copy KRSs from *L*. *loa* and *S*. *mansoni* can be effectively inhibited by cladosporin with more than 60 fold better binding than for the human counterpart enzyme. Crystal structure of *L*. *loa* KRS bound to cladosporin and L-lysine shows key interacting and selectivity residues. This work hence provides a platform for structure-guided derivatization of cladosporin-based compounds for drug development against these neglected diseases.

## Introduction

The worm parasites *Loa loa* (*Ll*) and *Schistosoma mansoni* (*Sm*) are causative agents of loiasis and schistosomiasis, respectively [1, 2]. *L*. *loa* is a member of the nematode phyla that infects ~13 million people every year in west and central Africa causing notable morbidity, disability and socioeconomic loss [2–4]. *L*. *loa* larvae are transferred to humans after the bite of infected deerfly vector (*Chrysops* spp.). These larvae slowly develop into mature adult parasites that traverse through various tissues and manifest angioedema, endomyocardial fibrosis, eosinophilia, encephalitis and nephropathy [2–5]. Their migration across eye conjunctiva has led to the common term of African eye worm [2–5]. Adults produce microfilariae by sexual reproduction and are re-circulated by flies during another blood meal [2–5]. These microfilariae then develop into infective larvae inside the fly [2, 3, 5]. Loiasis can be treated by the WHO recommended first line treatment of diethylcarbamazine or administration of alternative drugs like ivermectin and albendazole [3, 6]. These treatments, however, are not always easily applicable and pose life threatening risks [3, 6]. In contrast with Loiasis, schistosomiasis is a deadly neglected tropical disease that affects ~210 million people and kills >200,000 each year [2, 7, 8]. Schistosomiasis burden is mainly concentrated in the sub-Saharan Africa with highest prevalence in children and adults [2, 7, 8]. Human schistosomiasis is caused by three major Schistosoma species of platyhelminths phylum—*S*. *mansoni*, *S*. *japonicum and S*. *haematobium* [2, 7, 8]. These blood flukes complete their life cycle by shuttling between human and snail hosts. Adult *S*. *mansoni* reside in human vasculature and produce plentiful of eggs daily that are either excreted or deposited in the host liver. These events can trigger immune-mediated granuloma formation, hepatosplenism and periportal fibrosis leading to fatality [1, 2, 7, 8]. Single dose of praziquantel (PZQ) is almost entirely used for treatment and control of schistosomiasis, but this mass monodrug therapy may lead to drug resistance [2, 7, 8]. Additionally, the drug target for praziquantel remains unknown, which can hamper attempts to rationally design and synthesize second-generation drugs based on PZQ. Hence, both Loiasis and Schistosomiasis require discovery and validation of new druggable targets as well as insights into novel chemical scaffolds that can be used for drug development.

Others and we have shown that targeting of aminoacyl-tRNA synthetases (aaRSs) from infectious agents that cause malaria, toxoplasmosis, bacterial infections, fungal infections and leishmaniasis can be valuable [9–24]. The aaRSs control protein biosynthesis pathways by allowing pairing of cognate tRNA with amino acids [25]. Usually a cellular translational compartment contains 20 aaRSs, and depending on shared sequence motifs and topology in catalytic domains these aaRSs are divided into two classes. Class I enzymes contain the ATP binding motifs HIGH and KMSKS (Rossmann fold) while three conserved sequence motifs called 1, 2 and 3 are the characteristic of class II enzymes [25, 26]. Lysyl-tRNA synthetase (KRS) couples L-lysine to cognate tRNAs, and is the only aaRS that has evolved in different organisms to fall in both class I and II. While eukaryotes and most prokaryotes contain class II KRS, some bacteria and archaea contain class I [25, 27, 28]. The aaRSs can perform many non-canonical functions, and these have been documented for human as well as parasitic aaRSs [14, 29]. KRSs from many organisms, including the malaria parasite *P*. *falciparum*, have also been reported to synthesize signaling molecules like diadenosine polyphosphates (Ap4A, Ap5A) that modulate variety of cellular functionalities such as DNA replication, gene expression and ion channel regulation to mention a few [18, 22, 30, 31]. Crystal structures and functional analyses of human cytoplasmic KRS have shown that this enzyme can exist in tetrameric and dimeric forms, where the tetrameric form is bound to multi-synthetase complex and is translationally active, and the dimeric form can participate in transcription regulation and may have cytokine-like functions [32, 33]. Thus, determining the oligomeric status of KRSs is of key importance in understanding their functionality and mechanism. Previous reports on the malaria parasite *Plasmodium falciparum* (*Pf*) KRS showed that this dimeric protein is inhibited by the fungal secondary metabolite cladosporin with high potency [15, 22, 34]. Cladosporin targets malaria in both blood and liver stages with IC_50_ values below 100 nM [34]. This antimalarial effect is highly selective and mammalian cells are protected as assessed by cytotoxicity assays.

In an effort to understand the protein translation components responsible for supplying charged tRNAs for ribosomal protein synthesis within *L*. *loa* and *S*. *mansoni*, we first cataloged all their aaRSs and associated protein factors. We noted that *L*. *loa* and *S*. *mansoni* KRSs are present as single copy in both parasites. We discovered that cladosporin is a very potent inhibitor of *L*. *loa* and *S*. *mansoni* KRSs, and has enzyme inhibitory IC_50_ values in low nanomolar ranges (~52 nM and ~97 nM respectively). We provide the X-ray co-crystal structure of this drug bound *L*. *loa* KRS to demonstrate its binding mechanism and selectivity. Our proof-of-concept data on pathogen aaRSs predicts that targeting of other schistosome members and trypanosomes should also be feasible using the same chemical scaffold.

## Results

### The *L*. *loa* and *S*. *mansoni* aaRSs

Usually one aaRS is required per amino acid and thus a complete set of 20 aaRSs is necessary for protein translation in any cellular context when alternate pathways for producing charged tRNAs are not available [25, 26]. We thus looked for all sets of aaRSs in *L*. *loa* and *S*. *mansoni* genomes. *L*. *loa* genome encodes 35 putative aaRSs along with 5 accessory proteins–and this set likely fulfills aminoacylation requirements of its two translational chambers of cytoplasm and mitochondria (S1 Table) [35, 36]. A careful analysis of the predicted cellular distribution shows that 19 aaRSs are likely to be localized in the cytoplasm with an absence of KRS (S1 Table). Gene structure suggests that the single copy *Ll*KRS absence from the cytoplasm as it contains a predicted mitochondrial N-terminal signal sequence, but its sequence alignment, domain and motif analyses suggest it to be a eukaryotic-type protein, possibly dual localized. Putative aaRSs with specificity for 16 amino acids are present in *L*. *loa* mitochondria with 4 (cysteine, glutamine, glycine and threonine specific) missing aaRSs (S1 Table). Amongst these, the glutamine charged tRNA can be provided by indirect pathway involving putative mitochondrial glutamyl-tRNA amidotransferase [35, 36]. In comparison to *Loa loa*, *S*. *mansoni* contains a set of 19 cytoplasmic aaRSs with notable absence of glycine-specific aaRS (S2 Table). However, the mitochondrial GlyRS is a eukaryotic-type enzyme and its dual localization is likely, as has been reported in other organisms [13, 37]. The *S*. *mansoni* mitochondrial aaRSs set is deficients in GlnRS, HisRS and LysRS (S2 Table). Based on data from several laboratories including ours on aaRSs cellular distributions in eukaryotic pathogens, it is likely that the twin mechanisms of dual aaRs localizations and trafficking of charged tRNA across translational compartments are active in *L*. *loa* and *S*. *mansoni* as well, in order to provide all substrates required for protein translation in both compartments [13, 17, 37, 38].

### *L*. *loa* and *S*. *mansoni* KRSs are dimeric and enzymatically active

**Fig 1:**
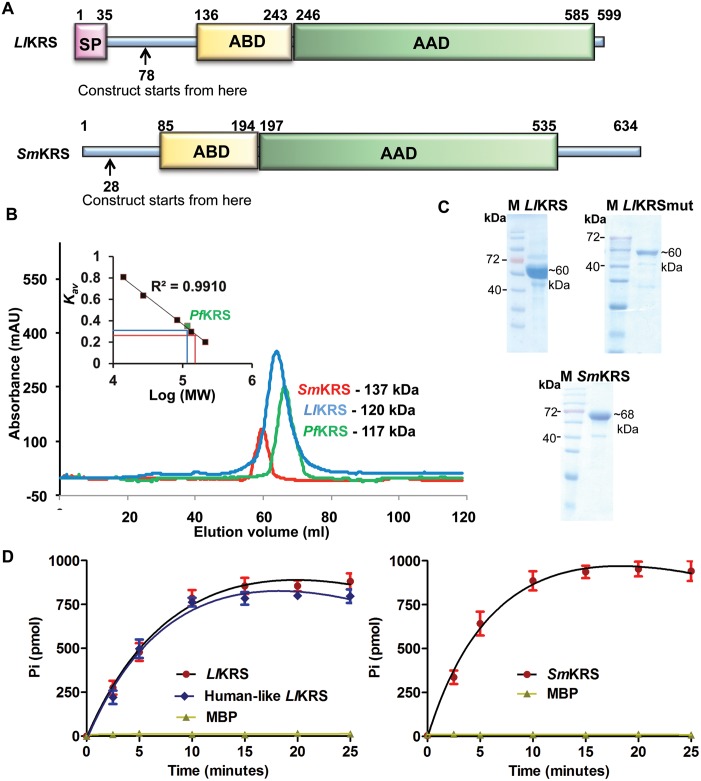
Domain structure, purification and activity of *L*. *loa* and *S*. *mansoni* KRSs. (A) Domain-wise architectures of *Ll*KRS and *Sm*KRS are shown. SP, ABD and AAD denote signal peptide (mitochondrial), anticodon binding domain and aminoacylation domains respectively. (B) GPC elution profile of purified *Ll*KRS (blue), *Sm*KRS (red) with *Pf*KRS (green). Comparison with standard markers shows that *Ll*KRS and *Sm*KRS elute at a size corresponding to dimeric states. No absorbance at tetrameric size was observed for either protein. (C) Final purified proteins on SDS-PAGE. *Ll*KRSmut denotes the human-like *Ll*KRS. (D) Time-dependent enzymatic activity assay for *Ll*KRS, human-like *Ll*KRS and *Sm*KRS proteins at constant substrate concentrations show that purified enzymes were active for aminoacylation.

*Ll*KRS and *Sm*KRS enzymes that contained the aminoacylation and anticodon binding domains were produced recombinantly in *E*. *coli* (Fig 1A). Our localization predictions and comparative sequence analysis hinted that the *Ll*KRS contained a mitochondrial signal peptide (1–35), while the *Sm*KRSs was predicted to be a cytoplasmic enzyme (Fig 1A). To assess the oligomer status of purified proteins, we performed gel permeation chromatography (GPC) experiments using a calibrated column with known standards [22]. Both wild type worm KRSs eluted at sizes corresponding to expected dimers in our GPC experiment, unlike the human counterpart that purportedly shows a tetrameric form (Fig 1B) [32]. Purified worm parasite proteins were used for enzyme assays using *Sm*tRNA^Lys^ and were found to be enzymatically active (Fig 1C and 1D).

### Cladosporin binding and inhibition of *L*. *loa* and *S*. *mansoni* KRSs

**Fig 2:**
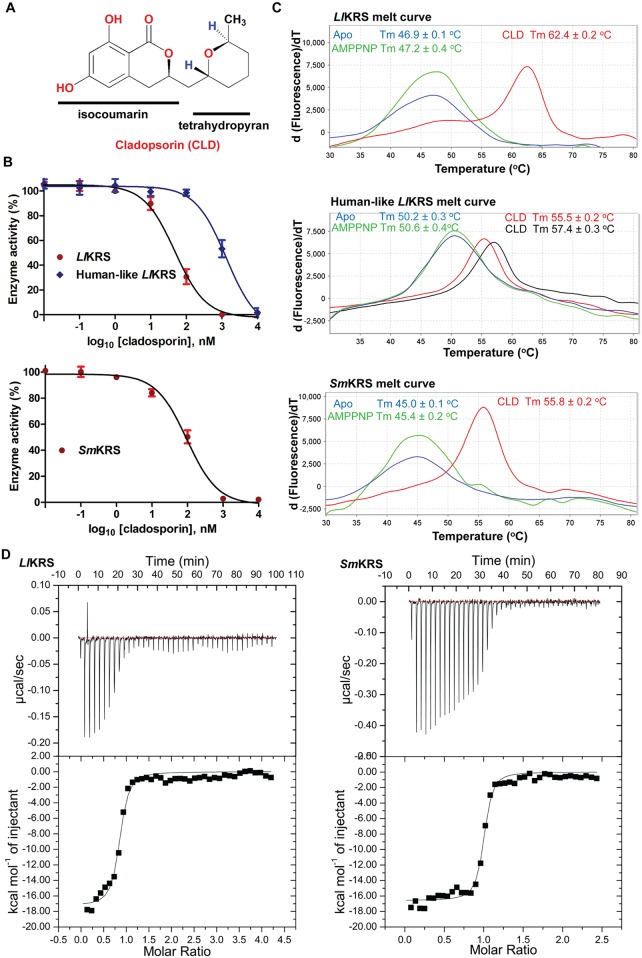
Cladosporin activity on worm KRSs. (A) Chemical structure of cladosporin. Cladosporin (CLD) is composed of a (6,8)-dihydroxyl- isocoumarin ring joined to tetrahydropyran group with a methyl moiety. (B) Inhibition of *Ll*KRS, human-like *Ll*KRS and *Sm*KRS by cladosporin in enzyme assays. Percentage enzyme activity as a function of increasing inhibitor concentration (log scale, 0.01 nM—10 μM) is plotted using non-linear regression. These results represent the mean of three independent experiments performed in triplicates. (C) Protein thermal shift profile of *Ll*KRS, *Sm*KRS and human-like *Ll*KRS (all three at 2 μM) in presence of cladosporin, or AMPPNP or without these two (but with L-lysine). The plot shows measured derivative Tm and data as plotted against fluorescence (arbitrary units) in y-axis and temperature in x-axis. Human-like *Ll*KRS thermal shift at two concentrations of cladosporin 20 μM (in red) and 200 μM (in black) is shown. Mean data from triplicates are presented. (D) Binding data using ITC. Cladosporin was titrated into the protein samples and K_d_ was determined using Microcal origin software.

Cladosporin is a 3,4-dihydro-6,8-dihydroxy-3-(6-methyl-tetrahydro-2H-pyran-2-yl) compound that mimics the adenosine part of ATP (Fig 2A). To test the activity inhibition and IC_50_ values of wild type *Sm*KRS and *Ll*KRS, enzyme assays were performed in presence of cladosporin. Human-like *Ll*KRS V329Q/S346T mutant protein was also produced by taking cues from previous reports and structural data analysis in this work (see next sections). The drug showed concentration-dependent enzymatic inhibition and revealed IC_50_ values of 52 nM and 97 nM for wild type *Ll*KRS and *Sm*KRS respectively, while a significantly higher IC_50_ value of 1370 nM was observed for the human-like *Ll*KRS mutant protein (Fig 2B). We also performed protein thermal shift assays to determine the binding of cladosporin to human-like *Ll*KRS and wild type worm KRSs in presence of L-lysine and (a) either no ligand, or (b) with non-hydrolysable ATP analogue (adenosine 5’-(β, γ-imido) triphosphate (AMPPNP)), or (c) with cladosporin in equal micromolar amounts. Data indicated that AMPPNP in a 10:1 molar ratio to KRSs was able to induce a small shift of ~0.3°C, ~0.4°C and ~0.4°C for *Ll*KRS, *Sm*KRS and for human-like *Ll*KRS respectively indicating very weak binding (Fig 2C). As expected, cladosporin when used in ten-fold higher molar concentration (20uM cladosporin) relative to KRSs (2 μM) was able to induce substantial shifts of 15.5°C and 10.8°C in both *Ll*KRS and *Sm*KRS respectively, indicating high affinity interactions with parasitic KRSs (Fig 2C). On the other hand, significantly smaller shift of 5.3 and 7.2°C were observed when human-like *Ll*KRS (2 μM) was incubated with ten fold (20 μM) or even hundred fold higher (200 μM) concentrations of cladosporin, indicating much poorer binding of cladosporin to the mutant *Ll*KRS (Fig 2C). To determine the binding affinity of cladosporin, we performed ITC experiments and discovered K_d_ values of 45.2 ± 8.4 nM and 62.8 ± 7.8 nM for *Ll*KRS and *Sm*KRS respectively (Fig 2D). Similar changes in binding enthalpy (ΔH) and entropy (TΔS) factors indicated conserved mechanism of worm KRS-cladosporin complexation (Table 1). Together, our enzyme inhibition, TSA and ITC data demonstrate strong affinity of cladosporin for these worm KRSs, and validate the potential of cladosporin to selectively bind parasitic KRSs over human counterpart where its affinity is relatively poor (3.3 μM) [15, 39].

**Table 1:** Isothermal titration calorimetry data showing strength of cladosporin binding.

Protein	Temperature°C	ΔH (cal/mol)	ΔS (cal/mol/deg)	n Value (one site model)	K_d_ (nM)
*Ll*KRS	30	-17280 ± 396.5	-24.9	0.821 ± 0.01	45.2 ± 8.4
*Sm*KRS	30	-16590 ± 201.3	-21.7	0.979 ±0.00	62.8 ± 7.8

### Structure of *Ll*KRS-CLD-K complex

**Fig 3:**
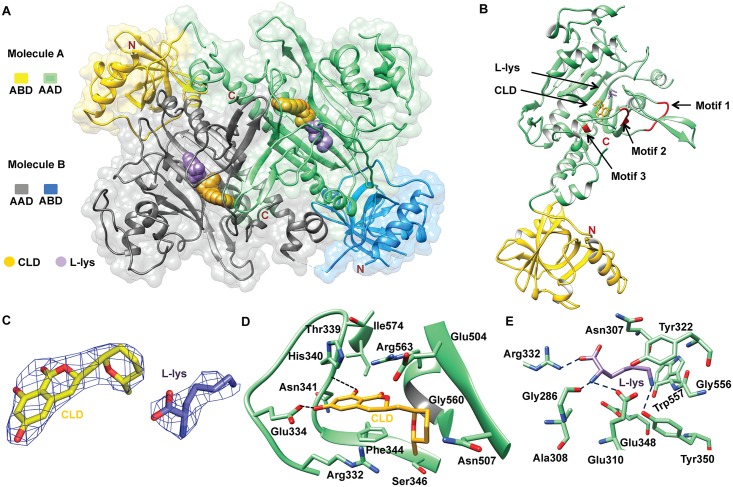
*Ll*KRS-CLD-K complex structure and interactions. (A) Dimer of *Ll*KRS with bound cladosporin (CLD) and L-lysine (L-lys). Two monomers are denoted as molecules A and B. The anticodon binding domain (ABD), aminoacylation domain (AAD), CLD and L-lysine are depicted. (B) *Ll*KRS monomer with bound cladosporin, L-lysine and motifs 1, 2 and 3 are highlighted in red. (C) View of experimental electron density at 1.2 σ (3.3 Å data) for cladosporin and L-lysine in *Ll*KRS-K-CLD structure. (D) Cladosporin-interacting residues in binding pocket are shown. Phe344, Arg563 and His340 stack with the isocoumarin moiety of cladosporin. Hydroxyl groups from isocoumarin moiety form hydrogen bonds with Glu334 and Asn341. Methyl group joined to the THP ring points towards Ser346. (E) The bound L-lysine in active site is shown.

To understand the atomic basis of KRS-cladosporin binding, we solved the crystal structure *L*. *loa* KRS in complex with cladosporin (CLD) and L-lysine (K). Crystal packing analysis showed two dimers of *Ll*KRS in the crystallographic asymmetric unit, validating our GPC results on recombinant *Ll*KRS (Figs 3A and 1B, Table 2). *Ll*KRS folds into a canonical eukaryotic KRS and contains N-terminal OB fold anticodon binding domain and a C-terminal catalytic domain (Fig 3B). The signature motifs 1, 2 and 3 present in the catalytic domain are also conserved (Fig 3B). Cladosporin fits into the ATP binding site in *Ll*KRS and interacts with most of the residues that accommodate adenosine moiety of ATP (Fig 3C and 3D). The isocoumarin ring of cladosporin is stabilized mainly by π-π staking with Phe344, T-stacking with His-340 and hydrogen bondings with Asn341 backbone and Glu334 (Fig 3D). In addition, guanidine group from Arg563 and Arg332 also stabilize the isocoumarin moiety (Fig 3D). Gly560 provides hydrophobic support to tetrahydropyran ring (THP) whereas the Ser346 lends suitable space for its methyl moeity. The L-lysine binds in the inner region of active site pocket and is accommodated by series of hydrogen bondings with protein atoms (Fig 3E).

**Table 2:** Data collection and refinement settings.

PDB code	5HGQ
Space group	P2_1_2_1_2_1_
Unit cell dimensions (Å,°)	a = 120.16, b = 147.35, c = 160.94
Molecules in ASU	4
Resolution range (Å)	50.00–3.30 (3.36–3.30)
Unique reflections	40375 (1996)
Completeness (%)	91.9 (91.6)
I/σ(I)	2.9 (0.7)
R_merge_ ^b^	0.239 (0.820)
Redundancy	3.3 (3.1)
Solvent content (%)	66
***Refinement***	
Mean B factor protein	43
R-factor/R_free_ (%) ^c^	25.3/29.1
rmsd^d^ in bond lengths (Å)	0.004
rmsd in bond angles (°)	0.917
No. of protein atoms/ASU	14637
No. of water molecules/ASU	7
Ligand molecules	3
***Ramachandran plot***	
Ramachandran favored (%)	95.8
Ramachandran outliers (%)	0.1

^*a*^Values in parentheses are for the highest resolution shell.

^*b*^Rmerge = ∑∑|Ihkl-Ihkl(j)|/ ∑∑Ihkl, where Ihkl(j) is the observed intensity and Ihkl is the final average intensity value.

^*c*^Rwork = ∑∑||Fobs|-|Fcalc||/∑|Fobs| and Rfree = ∑||Fobs|-|Fcalc||/∑|Fobs|, where all reflections belong to a test set of 5 % randomly selected data.

^*d*^ Root-mean square-deviation from ideal value.

### Comparisons with human KRS

**Fig 4:**
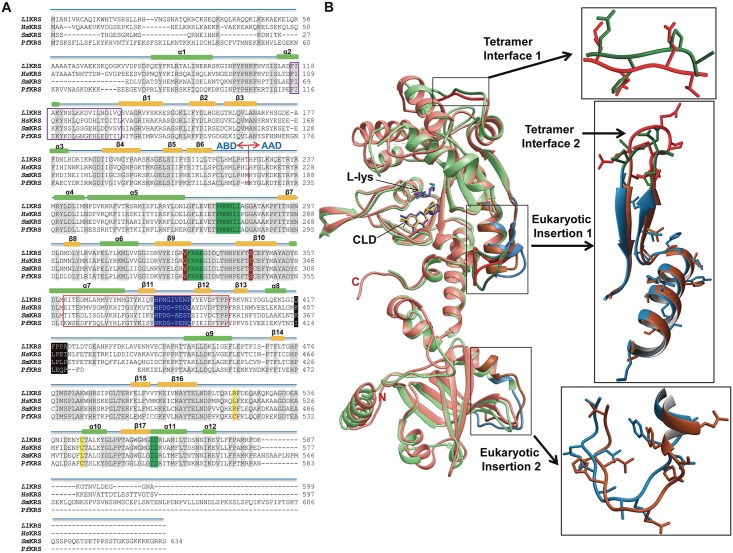
Comparisons with *HsKRS* structure. (A) Sequence alignment of *Ll*KRS, *Sm*KRS, *HsKRS* and *Pf*KRS. Anticodon binding domain (ABD) and aminoacylation domain (AAD) sequences along with class II motifs 1, 2 and 3 are shown (in green). Eukaryotic insertions 1 and 2 are shown in red and purple boxes respectively. *HsKRS* tetramer interface 1 is highlighted in black and interface 2 is in blue. Cladosporin-selectivity residues Ser346 and Val329 are highlighted in brown. Disulfide-bonded cysteines (Cys517 and Cys540) in *Pf*KRS and the orthologous residues in others are highlighted in yellow. (B) Cladosporin (CLD) and L-lysine (L-lys) bound *HsKRS* (4YCU) (in salmon) and *Ll*KRS (green) PDBs are superimposed. Architectural differences in eukaryotic insertions and at *HsKRS* tetramer interface are shown.

**Fig 5:**
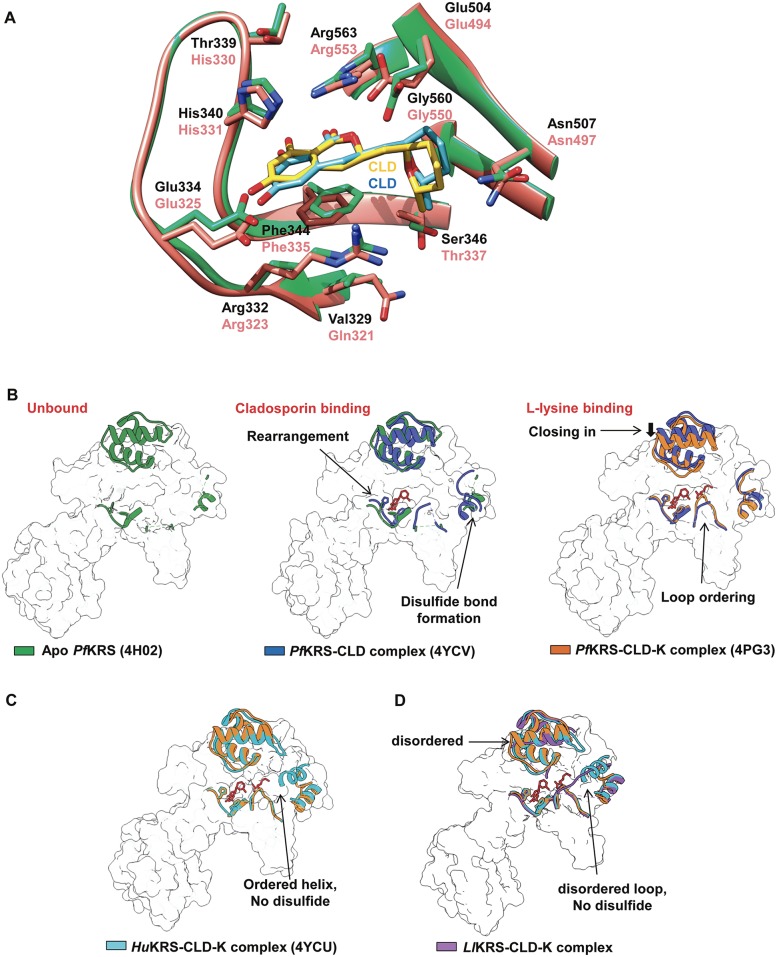
Cladosporin-binding mechanism of KRSs. (A) Cladosporin binding in *HsKRS* (salmon) and LlKRS (green). Residues Ser346 and Val329 are replaced by larger Thr337 and Glu504 in *HsKRS*. Cladosporin bound to *Hs*KRS structure is in blue and to *Ll*KRS is in yellow. (B) Structural changes in apo-*Pf*KRS (green) induced by cladosporin (CLD) and lysine (L-lys) individually are shown in blue and orange respectively. Cladosporin binding induces closing-in of the loop that contains motif 2, with rotameric adjustments in motif 2 residues Phe342, His338 and Arg559 (S1 Movie). This is accompanied by disulfide bond formation in the disordered loop (blue). L-lysine binding further induces a closing-in of mobile element present at roof of active site pocket and stabilization of the loop residues 580–590. The final *Pf*KRS-CLD-K complex with all four major transitions is shown in orange. (C) *Hs*KRS-CLD-K complex (cyan) overall conformation is similar to *Pf*KRS-CLD-K complex (orange). The disulfide-stabilized loop is in an ordered helix in *Hs*KRS. (D) *Ll*KRS differs from previous KRS-cladosporin structures in that the incoming mobile roof and the disulfide regions are both disordered. Hence, cladosporin selectivity for *P*. *falciparum*, *L*. *loa* and perhaps *S*. *mansoni* KRSs is likely driven by the conserved residues Ser and Val that distally line active site pockets in these pathogen KRSs.

A comprehensive sequence alignment of cladosporin-sensitive pathogen KRSs like *Pf*KRS, *Sm*KRS, *Ll*KRS was used to map drug-binding residues in the active sites of these KRSs (Fig 4A and 4B). *Hs*KRS (PDB: 4YCU) and *Ll*KRS share ~66% overall sequence identity and show r.m.s.d. of 1.88 Å in their cladosporin-bound forms for 463 C^α^ atoms. The earlier reported human tetramer enzyme (PDB: 3BJU) and our observed *Ll*KRS dimer show differences in the amino acid sequences and topology of tetramer interface regions 1 and 2 (Fig 4A and 4B) [32]. Despite the comparatively conserved eukaryotic insertion 1 in *Ll*KRS, the sequence and structural differences in tetramer interface region within it appear to have endowed only dimeric conformation to the worm KRSs (Fig 4A and 4B). Interestingly, *Ll*KRS is sequence-wise and in architectural terms (r.m.s.d.) closer to *Hs*KRS than the *Pf*KRS (54% sequence identity and 2.22 Å r.m.s.d. with *Ll*KRS for 455 C^α^ atoms), and yet possesses cladosporin sensitivity like *Pf*KRS. Availability of recent cladosporin-bound *Hs*KRS structure provided an opportunity for us to compare the human and worm enzymes. The active site region and binding mechanism of cladosporin for both *Ll*KRS and *Hs*KRS is remarkably similar, with only difference of Ser346/Thr337 (*Ll*/*Hs*) and distant Val329/Gln321 (*Ll*/*Hs*) substitutions (Fig 5A). It is clear that the residues Ser346 and Val329 provide extra space for accommodating the methyl moiety of THP ring, and they thus contribute to species selectivity [15, 34, 39]. *Pf*KRS is currently the best-studied model for understanding cladosporin-binding mechanism, and in addition to two selective residues, many other structural aspects of malaria KRS that contribute to selectivity have become apparent during recent structural investigations by our group and from others [15, 22, 39]. To understand the *Ll*KRS cladosporin binding and specificity, we analyzed it in backdrop of known *Plasmodium* and human KRS structures [15, 22, 39]. In *Pf*KRS, binding of cladosporin induces a loop movement (near motif II) of approximately ~2.4 Å towards the active site, and rearrangements of His338 (*Ll*His331), Phe342 (*Ll*Phe335) and Arg559 (*Ll*Arg553) occur to accommodate isocoumarin moiety of cladosporin (Fig 5B and S1 Movie). These events coincide with formation of disulfide bond in a disordered loop region of *Pf*KRS (Fig 5B). The L-lysine induces an inward mobility in the active site roof region and also stabilizes a disordered loop (Fig 5B). All four major transitions are present in the *Pf*KRS-CLD-K complex, and the recent crystal structure of *Hs*KRS-CLD-K also shows a structural state similar to *Pf*KRS (r.m.s.d. 1.43 Å for 490 Cα atoms) [15, 39] (Fig 5C). The L-lysine induced changes have recently been proposed to be the main factor driving cladosporin species selectivity (Fig 5B) [39]. To address this, we compared cladosporin and L-lysine bound *Ll*KRS structure to the already available *Hs*KRS and *Pf*KRS cladosporin-bound structures. We found that a helix in L-lysine-induced mobile body is disordered in *Ll*KRS (Fig 5D). Additionally, the stable helix (in case of *Hs*KRS) or the disulfide stabilized loop (in case of *Pf*KRS) are also absent in *Ll*KRS and that this region is disordered (Fig 5D). These structural observations hence support the observation that most likely the conserved pair of Val329 and Ser346 found in pathogen KRSs drive cladosporin selectivity.

## Discussion

Helminths represent some of the most prevalent neglected tropical disease parasites, and schistosomiasis likely ranks just below malaria as a cause of misery in context of public health [2, 7, 40]. The currently available monodrug treatment of schistosomiasis using praziquantel poses threat of possible drug resistance [2, 7, 8, 40]. On the other hand, loiasis is prevalent in rainforest and low socioeconomic regions, and has gained prominence in recent years due to adverse effects of drug treatments during co-endemicity with other filarial pathogens [3, 6]. Research efforts directed at understanding vital cellular processes such as protein translation machinery can hugely benefit drug discovery initiatives, especially given the promise of utility in context of other infectious diseases like malaria. This is especially of benefit to neglected tropical disease research, where efforts to develop drugs needs to be cost effective. Prompted by these concerns, we sought to dissect worm aaRSs that are responsible for protein translation in these organisms. In this report, we have provided a comprehensive overview of the aaRS distributions in genomes of *L*. *loa* and *S*. *mansoni*. In both these organisms, it is likely that aaRSs fulfill translational requirements in two cellular compartments of mitochondria and cytoplasm by evolutionarily successful mechanisms of aaRS dual localization, indirect aminoacylation pathways and trafficking of charged tRNAs [13, 17, 37, 38]. Further, the presence of single copy KRSs in both pathogens presents a lucrative opportunity to target this enzyme so as to dismantle protein synthesis process in two translational compartments simultaneously.

**Fig 6:**
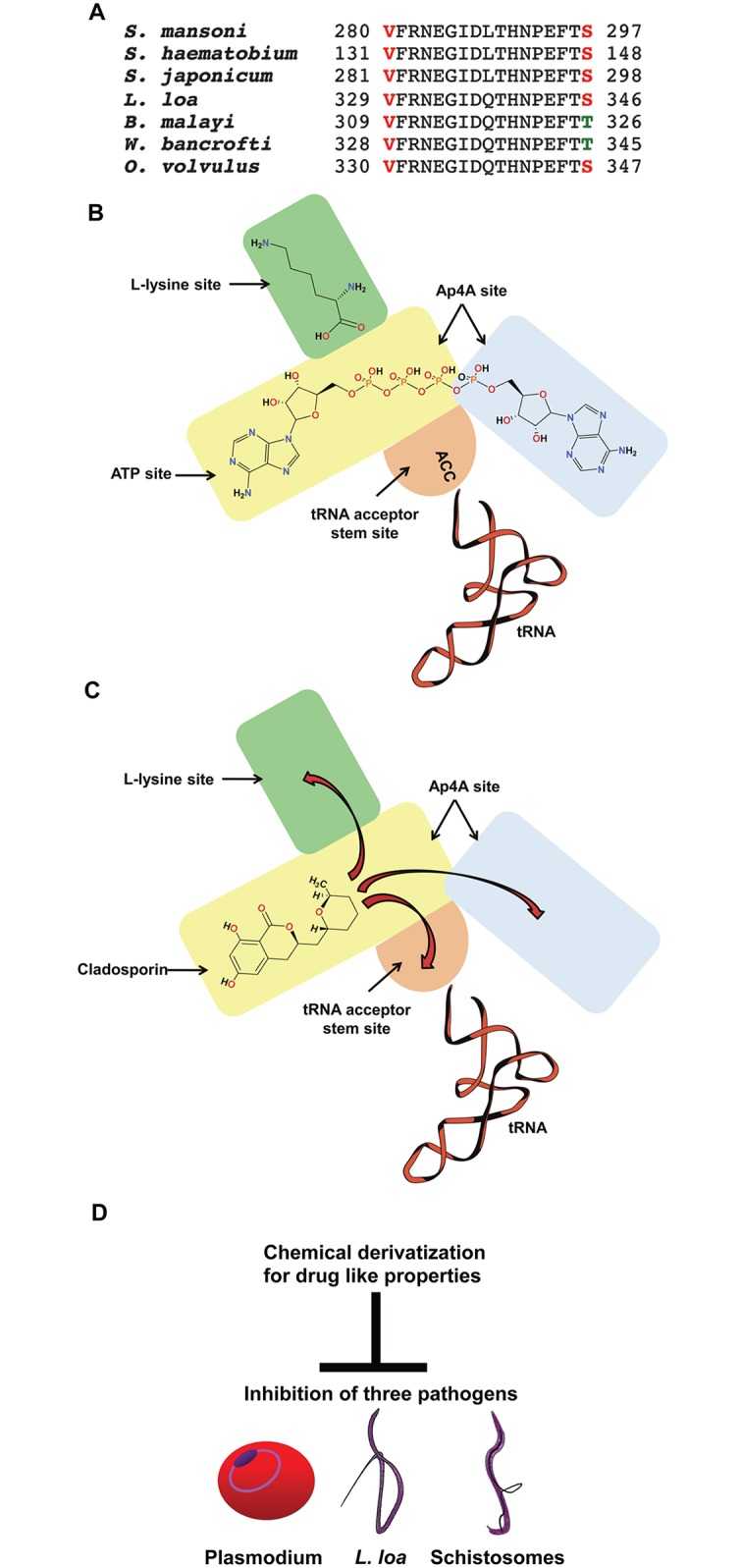
Cladosporin derivatization strategy. (A) Sequence alignment of schistosome and *L*. *loa*-related nematodes are shown. (B) Cartoon representation of the general reaction centers with substrates in KRS is shown. KRSs bind to ATP, L-lysine and CCA acceptor stem region of tRNA to carry aminoacylation reaction. Ap4A can be formed by KRSs as well. (C) Cladosporin derivatization to improve its ADME properties may focus on sites indicated with red arrows, or stereoisomeric alterations. (D) Cladosporin-based libraries may be useful across a spectrum of pathogens where KRS active sites and selectivity residues are conserved.

We additionally found that other members of Schistosoma genus like *S*. *japonicum* (GeneBank: CAX83109.1) and S. *haematobium (*GeneBank: KGB31491.1) also possess the conserved cladosporin-sensitive motif and hence could be targeted by cladosporin via their KRSs (Fig 6A). Interestingly though, sequence analyses show that *L*. *loa* and *O*. *volvulus* could be specific targets amongst pathogenic filarial nematodes (OVOC_0000240101-mRNA-1) (Fig 6A). Sequence differences in *L*. *loa*, *W*. *bancrofti* (GeneBank: EJW79634.1) and Brugia malayi (GeneBank: CDP92701.1) KRSs suggest that the latter two might be less sensitive to cladosporin, hence providing an opportunity to selectively target *L*. *loa* and *O*. *volvulus* during co-infections (Fig 6A). Poor bioavailability is the main limitation in development of cladosporin as a lead molecule [34]. Chemical synthesis protocols for cladosporin are now available which can aid in structure-guided rational synthesis of more drug-like cladosporin derivatives [41]. Apart from loiasis and schistosomiasis, suitable derivatization of cladosporin for better ADME (absorption, distribution, metabolism, and excretion) properties will be highly valuable for drug development against host of parasitic infections including malaria and feasibly trypanosomosis (given the conservation in active site residues that recognize cladosporin, Fig 6B and 6C) [15]. Hence, cladosporin-based small molecular libraries could be very good starting points for cell-based and phenotypic screening against a number of eukaryotic pathogens. The presented data here therefore provide new avenues for novel drug development against parasitic worm diseases and highlights numerous potential aaRS targets that can now be exploited.

## Materials and Methods

### Identification and annotation of aaRSs from *L*. *loa and S*. *mansoni*

The aaRSs and accessory proteins were identified using HMM-search tool in the HMMER web server (http://www.ebi.ac.uk/Tools/hmmer/) by restricting the taxonomy against *L*. *loa* and *S*. *mansoni* and with an E-value cut-off of 0.01. Pfam IDs of aaRSs and accessory proteins were used in the HMMER based search. Each hit was verified further by sequence, domain and motif analyses using SMART [42], CD-search [43] and superfamily servers [44]. Localizations were predicted using online servers MitoProt (mitochondrial localization- http://ihg.gsf.de/ihg/mitoprot.html) and NucPred (nuclear localization) [45]. Available mitochondrial localization prediction softwares are trained on non-helminthes organisms and thus sequence alignments with respective validated mitochondrial aaRSs (from NCBI) were also used to identify prokaryotic/mitochondrial type aaRSs. Mitochondrial/prokaryotic type aaRSs found in our analysis, but without the predicted mitochondrial signal sequence were also assigned as putatively mitochondrial. Splice variants from single gene and any atypical aaRSs found in Uniprot [46] were verified via EnsemblMetazoa transcript database (http://metazoa.ensembl.org/index.html). Protein sequences for related worm parasites were obtained from *Ll*KRS or *Sm*KRS protein blast while *O*. *volvulus* KRS (OVOC_0000240101-mRNA-1) sequence was obtained from *Ll*KRS protein blast in http://www.sanger.ac.uk/.

### Molecular cloning, expression and purification

*Ll*KRS and *Sm*KRS protein sequences were aligned against *Pf*KRS PDB sequence (4PG3) to identify N-terminal sequences of unknown functions or a possible signal sequence, i.e. 1 to 27 for *Sm*KRS and 1 to 77 for the *Ll*KRS. Gene sequences encoding protein residues 28–634 of *Sm*KRS and 78–599 of *Ll*KRS were designed for expression in *Escherichia coli* and subcloned into pETM-41 vector using NcoI and KpnI restriction sites. Human-like *Ll*KRS V329Q / S346T mutant was created by site directed mutagenesis in two positions V329 to Q and S346 to T. Cloning, expression and purification of the human-like *Ll*KRS protein was performed as for the wild type *Ll*KRS. Protein expression for wild type KRSs and human like *Ll*KRS was induced by adding 0.5 mM isopropyl -d-1-thiogalactopyranoside (IPTG) to cells grown till OD_600_ of 0.6–0.8 at 37°C. These cells were grown for 20 h post-induction at 18°C. Bacterial cells were harvested by centrifugation at 5000 g for 15 min and the bacterial pellets were suspended in a buffer consisting of 50 mM Tris–HCl (pH 8.0), 200 mM NaCl, 10 mM beta-mercaptoethanol (βMe), 15%(v/v) glycerol, 0.1 mg ml^-1^ lysozyme and 1 mM phenylmethylsulfonyl fluoride (PMSF). Cells were lysed by sonication and cleared by centrifugation at 20,000 g for 45 min. The cleared supernatants were applied onto amylose beads (GE Healthcare). All three MBP-KRS fusion proteins were eluted with 10 mM maltose in 50 mM Tris–HCl (pH 8.0), 200 mM NaCl, 10 mM βMe, 1 mM DTT, 0.5 mM EDTA. The MBP tag was removed by incubation of eluted pure fractions with TEV protease at 293 K for 24 h. Wild type and mutant cleaved worm KRSs were concentrated using a 10 kDa cutoff Centricon centrifugal device (Millipore) and were purified by gel-filtration chromatography on a GE HiLoad 60/600 Superdex column. Pure fractions were checked by SDS–PAGE and pooled for crystallization. Before crystallization, the wild type *Ll*KRS was concentrated to 10 mg/ml (A280, extinction coefficient- 46760 M^-1^ cm^-1^) and stored at -80°C. To determine the oligomer status, high molecular weight calibration standards (GE Healthcare) and purified proteins were run on GPC column using protein buffer mobile phase at flow rate of 0.5 ml/ min. Molecular weights of *Ll*KRS and *Sm*KRS were estimated from their elution profiles by plotting log molecular weight (X-axis) against partition coefficient (*K*_*av*_, Y-axis) for known standards.

### Aminoacylation assays

*Sm*tRNA^Lys^ was synthesized using an *in-vitro* transcription method described earlier with minor modifications [47]. A double stranded DNA sequence (TCAGTAGCTG AGTGGATAAT GCGA TGGCGT TTTAAGCGAA CGGTACTGGG TTCGAGTCCCAGAGTGA*ACCA)* encoding cytoplasmic *Sm*tRNA^Lys^ (GeneDB: SmtRNA_01463_Lys_TTT.1.1) containing 5’ T7 RNA polymerase promoter, CCA sequence at 3’ (in italics) and 2’-*O* methyl substitution in last two nucleotides of antisense strand was purchased (Sigma). This sequence was transcribed using T7 quick high yield RNA synthesis kit (NEB) at 37°C for 16 h according to manufacturer’s instructions. DNA template was removed by DNase (10U/ml) treatment for 3 h in ice followed by addition of EDTA (50 mM). Transcripts were extracted using phenol/CH_3_Cl/isoamyl alcohol (25:24:1) and ethanol precipitation method and reconstituted in 20 mM HEPES, 5 mM EDTA. Samples were further purified using DEAE column (binding buffer 100 mM HEPES-KOH, 12 mM MgCl_2_, 200 mM NaCl (pH 7.5) and elution buffer 100 mM HEPES-KOH, 12 mM MgCl_2_, 800 mM NaCl (pH 7.5)). Fractions containing tRNA were ethanol precipitated, their quality was checked on SDS-urea PAGE and samples were resuspended in 5 mM HEPES-KOH, 1 mM EDTA for storage at -80°C at a concentration of 50 μM. Aminoacylation and enzyme inhibition assays for both KRSs were performed as described elsewhere [17, 48]. *Sm*tRNA^Lys^ was refolded prior to enzyme assays by heating at 70°C for 10 minutes followed by addition of 10 mM MgCl_2_ and slow cooling to room temperature. Aminoacylation buffer for both worm KRSs contained 30 mM HEPES (pH 7.5), 150 mM NaCl, 30 mM KCl, 50 mM MgCl_2_, 1mM DTT, 100 μM ATP, 500 μM L-lysine, 18 μM *Sm*tRNA^Lys^, 2 U/ml *E*. *coli* inorganic pyrophosphatase (NEB) and 500 nM recombinant *Sm*KRS or *Ll*KRS protein at 37°C. Reaction at different time points was stopped by addition of 40 mM EDTA followed by transfer to ice. Recombinant maltose binding protein (MBP) was used as a control. Cladosporin inhibition assays were performed using inhibitor concentrations in log values ranging from 0.01 nM to 10 μM in the assay buffer.

### Thermal shift assays

Protein melt curve assays for both worm KRSs were performed as reported earlier [49]. Both KRSs were diluted in buffer containing 20 mM Tris (pH 8.0), 200 mM NaCl, 5 mM MgCl_2_, 1 mM L-lysine and 2 X SYPRO orange dye (Life Technologies). Total of 20 μM of each ligand AMPPNP (Sigma) and cladosporin (gifted by Bart Staker, SSGCID) was added separately to 2 μM KRS proteins and incubated in ice for 10 min. Ligand bound and unbound samples of both KRSs were heated from 20 to 96°C at a rate of 1°C min^-1^ and fluorescence signals were monitored by StepOnePlus quantitative real-time PCR system (Life Technologies). Human-like *Ll*KRS (2 μM) was tested in presence of 20 μM and 200 μM cladosporin concentrations to demonstrate weak binding even with higher inhibitor concentrations. Each curve was an average of three measurements and data were analyzed using thermal shift software (Life technologies). Samples without the addition of ligands were used to determine thermal melting profile of apo proteins. Cladosporin alone and AMPPNP alone in assay buffers, along with no protein controls were used and flat lines were observed for these fluorescence readings at all temperatures. Derivative Tm (melting temperature) was used for analysis.

### Isothermal Titration Calorimetry experiments (ITC)

ITC experiments were conducted at 30°C in the MicroCal ITC-200 apparatus (GE Healthcare) and results were fitted into graph using Microcal origin software. Both parasite KRSs were prepared in PBS (phosphate-buffered saline) pH 7.4 with 1 mM L-Lys and 2 mM MgCl_2_, and cladosporin was solubilized in the same buffer. Cladosporin at concentrations of 240 μM and 360 μM was titrated into 21 μM and 22.5 μM protein concentrations of *Sm*KRS and *Ll*KRS respectively. For *Ll*KRS, titrations consisted of 0.4 μl of first injection followed by 39 injections of 1 μl with 150 s intervals between injections. For *Sm*KRS, same titrations were performed with 120 s intervals. Titration of cladosporin in buffer alone was performed to determine the change in enthalpy caused by ligand dilution and then subtracted as background from the actual ligand-binding experiments. Limited protein precipitation with *Ll*KRS during our multiple ITC experiments (in various conditions for n-value optimization trials) was observed and possibly contributed to the lower n-value, which nonetheless is verified as value of 1 based on the crystal structure of the enzyme-drug complex we present here.

### Crystallization, data collection and structure determination

Crystallization trials for both *Sm*KRS and *Ll*KRS were performed and crystals of *Ll*KRS were obtained at 20°C by the hanging-drop vapor-diffusion method in 1:1 ratio of *Ll*KRS (10 mg ml^-1^) and mother liquor 20%(w/v) PEG 3350, 200 mM magnesium acetate and 10mM spermidine. Thin, plate-shaped crystal clusters soaked in cryoprotectant 20% glycerol were directly mounted in cooled nitrogen gas at 100 K. X-ray diffraction data were collected using a MAR CCD detector on beamline BM14 of the European Synchrotron Radiation Facility, Grenoble, France. A total of 150 images were collected with 1 min exposure and 1 oscillation per frame. The diffraction images were processed and scaled with HKL-2000 program suite [50]. The structure was solved using phaser-MR with *Hs*KRS as template (66% sequence identity, PDB: 3BJU) [51]. Initial model was built by AutoBuild in PHENIX [51] followed by multiple rounds of manual building using Coot [52] in combination with *phenix*.*refine* refinement cycles in PHENIX [51]. All structural superimpositions and preparation of figures was conducted using Chimera [53] and PyMol (http://www.pymol.org). Efforts to crystallize *Sm*KRS, though challenging, are ongoing.

## Supporting Information

S1 Table Putative *L*. *loa* aaRSs and their predicted localizations. Uniprot IDs of the predicted aaRSs in *L*. *loa* are shown. Single gene variants are shown in italics. (N) denotes the predicted nuclear localization. Putative cytoplasmic phenylalanyl-tRNA synthetase is a heterodimer and the subunits are denoted as (α) and (β) alongside their gene IDs. Three subunits of glutamyl-tRNA amidotransferase are denoted as (A), (B) and (C).
(DOCX)

S2 Table Putative *S*. *mansoni* aaRSs and their predicted localizations. Uniprot IDs of the predicted aaRSs in *S*. *mansoni* are shown. Single gene variants are shown in italics. (N) denotes the predicted nuclear localization. Putative cytoplasmic phenylalanyl-tRNA synthetase is a heterodimer and the subunits are denoted as (α) and (β) alongside their gene IDs. Two subunits of glutamyl-tRNA amidotransferase are denoted as (A) and (B).
(DOCX)

S1 Movie Morph movie for cladosporin (shown in yellow) binding to *Pf*KRS (shown in blue) is shown. In unbound state, Phe342 conformation disallows cladosporin stacking. Additionally, other active site residues are in an unfavorable non-binding conformation. Cladosporin selects for a conformation suitable for stacking and possibly induces rotameric adjustments in active site residues that together stabilize binding.
(AVI)
