# Supplementary material for: Protein Translation Enzyme lysyl-tRNA Synthetase Presents a New Target for Drug Development against Causative Agents of Loiasis and Schistosomiasis
Source: PLoS Negl Trop Dis. 2016 Nov 2;10(11):e0005084. doi: 10.1371/journal.pntd.0005084 (PMC5091859; doi:10.1371/journal.pntd.0005084)
Supplement: S1 Table — Uniprot IDs of the predicted aaRSs in L. loa are shown. Single gene variants are shown in italics. (N) denotes the predicted nuclear localization. Putative cytoplasmic phenylalanyl-tRNA synthetase is a heterodimer and the subunits are denoted as (α) and (β) alongside their gene IDs. Three subunits of glutamyl-tRNA amidotransferase are denoted as (A), (B) and (C). (DOCX) [file pntd.0005084.s001.docx]

**Supplementary table 1. Putative *L. loa* aaRSs and their predicted localizations.**

| **Protein Name** | **Mitochondria** | **Cytoplasm** |
| --- | --- | --- |
| **Class I** | | |
| Arginyl-tRNA synthetase | E1FN22_LOALO (N) | E1FIS4_LOALO |
| Cysteinyl-tRNA synthetase |  | *J0XF29_LOALO* (N)  *J0DVW8_LOALO* (N) |
| Glutamyl-tRNA synthetase | E1FQX8_LOALO (N)  *J0E0S4_LOALO*  *E1G2R2_LOALO* |  |
| Glutamyl-prolyl-tRNA synthetase  (bifunctional) |  | J0XI64_LOALO |
| Glutaminyl-tRNA synthetase |  | J0DVR6_LOALO |
| Isoleucyl-tRNA synthetase | J0XL30_LOALO | E1FJ74_LOALO |
| Leucyl-tRNA synthetase | *E1G182_LOALO*  *J0XME1_LOALO* | E1G239_LOALO  J0E169_LOALO |
| Methionyl-tRNA synthetase | E1FMW4_LOALO | E1FUL8_LOALO |
| Tryptophanyl-tRNA synthetase | J0XLM5_LOALO (N) | J0M4P5_LOALO (N) |
| Tyrosyl-tRNA synthetase | E1FTK6_LOALO (N) | E1GCJ4_LOALO |
| Valyl-tRNA synthetase | E1FHC1_LOALO | J0DNX0_LOALO |
| **Class II** | | |
| Alanyl-tRNA synthetase | E1G5W7_LOALO | E1FMK3_LOALO |
| Asparaginyl-trna synthetase | E1G6H4_LOALO | E1G569_LOALO |
| Aspartyl-tRNA synthetase | E1FTS8_LOALO  E1FTS7_LOALO | E1GCZ4_LOALO |
| Glycyl-tRNA synthetase |  | E1FYA2_LOALO |
| Histidyl-tRNA synthetase | J0DMD9_LOALO | E1FW59_LOALO |
| Lysyl-tRNA synthetase | E1FQP0_LOALO |  |
| Phenylalanyl-tRNA synthetase | E1FHC2_LOALO | E1FKW1_LOALO (α)  E1FZ98_LOALO (β) |
| Prolyl-tRNA synthase | J0XHC2_LOALO | J0DYT5_LOALO |
| Seryl-tRNA synthetase | E1FIK9_LOALO (N) | E1G0Z2_LOALO |
| Threonyl-tRNA synthetase |  | E1FXM7_LOALO |
| **Accessory proteins** | | |
| D tyrosyl-tRNA deacylase |  | E1FZM3_LOALO |
| P43 |  | E1FUL9_LOALO |
| L-seryl- tRNA (sec) kinase |  | E1FIY2_LOALO (N)  J0XH39_LOALO |
| O-phosphoseryl-tRNA(Sec) selenium  transferase |  | J0DRY6_LOALO |
| Glutamyl-tRNA amidotransferase | E1FIL4_LOALO (A)  E1FX42_LOALO (B)  GATC_LOALO (C) |  |
