# Supplementary material for: Protein Translation Enzyme lysyl-tRNA Synthetase Presents a New Target for Drug Development against Causative Agents of Loiasis and Schistosomiasis
Source: PLoS Negl Trop Dis. 2016 Nov 2;10(11):e0005084. doi: 10.1371/journal.pntd.0005084 (PMC5091859; doi:10.1371/journal.pntd.0005084)
Supplement: S2 Table — Uniprot IDs of the predicted aaRSs in S. mansoni are shown. Single gene variants are shown in italics. (N) denotes the predicted nuclear localization. Putative cytoplasmic phenylalanyl-tRNA synthetase is a heterodimer and the subunits are denoted as (α) and (β) alongside their gene IDs. Two subunits of glutamyl-tRNA amidotransferase are denoted as (A) and (B). (DOCX) [file pntd.0005084.s002.docx]

**Supplementary table 2. Putative *S. mansoni* aaRSs and their predicted localizations.**

| **Protein Name** | **Mitochondria** | **Cytoplasm** |
| --- | --- | --- |
| **Class I** | | |
| Arginyl-tRNA synthetase | G4VK38_SCHMA | G4V8B4_SCHMA |
| Cysteinyl-tRNA synthetase | *G4VAA6_SCHMA* (N) | *G4VAA5_SCHMA* (N) |
| Glutamyl-tRNA synthetase | G4V9R9_SCHMA (N) |  |
| Glutamyl-prolyl-tRNA synthetase (bifunctional) |  | *G4VLU4_SCHMA* (N) *G4VLU3_SCHMA* |
| Glutaminyl-tRNA synthetase |  | G4V8J0_SCHMA |
| Isoleucyl-tRNA synthetase | G4LVH7_SCHMA | G4VMZ5_SCHMA |
| Leucyl-tRNA synthetase | G4VDW6_SCHMA (N) | G4VAL2_SCHMA |
| Methionyl-tRNA synthetase | G4LZ29_SCHMA | G4VFQ2_SCHMA |
| Tryptophanyl-tRNA synthetase | G4LXR8_SCHMA (N) | G4VC69_SCHMA (N) |
| Tyrosyl-tRNA synthetase | G4M0H1_SCHMA | G4M0L7_SCHMA (N) |
| Valyl-tRNA synthetase | G4VS47_SCHMA (N) | G4M0N7_SCHMA |
| **Class II** | | |
| Alanyl-tRNA synthetase | G4VQH1_SCHMA (N) | G4VNT2_SCHMA |
| Asparaginyl-trna synthetase | G4VCE1_SCHMA | G4LWW1_SCHMA |
| Aspartyl-tRNA synthetase | C1M093_SCHMA  G4M1Q3_SCHMA | *G4VJ11_SCHMA*  *G4VJ12_SCHMA* |
| Glycyl-tRNA synthetase | G4VFP9_SCHMA (N) |  |
| Histidyl-tRNA synthetase |  | *G4VFX7_SCHMA*  *G4VFX6_SCHMA* |
| Lysyl-tRNA synthetase |  | *G4M0D9_SCHMA*  *G4M0E0_SCHMA* |
| Phenylalanyl-tRNA synthetase | G4VKS1_SCHMA | C4QNZ6_SCHMA (α)  G4LV70_SCHMA (β) (N) |
| Prolyl-tRNA synthase | G4VPF4_SCHMA |  |
| Seryl-tRNA synthetase | G4V5K7_SCHMA | G4VIZ9_SCHMA (N) |
| Threonyl-tRNA synthetase | G4V6P2_SCHMA (N) | G4V6P1_SCHMA |
| **Accessory Proteins** | | |
| D tyrosyl-tRNA deacylase |  | G4LVJ8_SCHMA |
| P43 |  | G4V705_SCHMA |
| O-phosphoseryl-tRNASec kinase |  | G4VB51_SCHMA (N) |
| L-seryl-tRNA (Sec) kinase |  | G4VH30_SCHMA |
| O-phosphoseryl-tRNA (Sec) selenium transferase |  | G4VL61_SCHMA (N) |
| Glutamyl-tRNA amidotransferase | G4LYL3_SCHMA (A)  G4LWC3_SCHMA (B) |  |
